# Unveiling the Local Atomic Arrangements in the Shear Band Regions of Metallic Glass

**DOI:** 10.1002/adma.202007267

**Published:** 2021-02-19

**Authors:** Xiaoke Mu, Mohammed Reda Chellali, Evgeniy Boltynjuk, Dmitry Gunderov, Ruslan Z. Valiev, Horst Hahn, Christian Kübel, Yulia Ivanisenko, Leonardo Velasco

**Affiliations:** ^1^ Institute of Nanotechnology Karlsruhe Institute of Technology Eggenstein‐Leopoldshafen 76344 Germany; ^2^ Saint Petersburg State University St. Petersburg 199034 Russia; ^3^ Institute of Molecule and Crystal Physics Ufa Federal Research Center RAS Ufa 450075 Russia; ^4^ Institute of Physics of Advanced Materials Ufa State Aviation Technical University Ufa 450008 Russia; ^5^ Joint Research Laboratory Nanomaterials Technische Universität Darmstadt Darmstadt 64206 Germany; ^6^ Karlsruhe Nano Micro Facility Karlsruhe Institute of Technology Eggenstein‐Leopoldshafen 76344 Germany

**Keywords:** atom probe tomography, bulk metallic glasses, pair distribution function, shear bands and affected zones, STEM pair distribution function (STEM‐PDF)

## Abstract

The prospective applications of metallic glasses are limited by their lack of ductility, attributed to shear banding inducing catastrophic failure. A concise depiction of the local atomic arrangement (local atomic packing and chemical short‐range order), induced by shear banding, is quintessential to understand the deformation mechanism, however still not clear. An explicit view of the complex interplay of local atomic structure and chemical environment is presented by mapping the atomic arrangements in shear bands (SBs) and in their vicinity in a deformed Vitreloy 105 metallic glass, using the scanning electron diffraction pair distribution function and atom probe tomography. The results experimentally prove that plastic deformation causes a reduction of geometrically favored polyhedral motifs. Localized motifs variations and antisymmetric (bond and chemical) segregation extend for several hundred nanometers from the SB, forming the shear band affected zones. Moreover, the variations within the SB are found both perpendicular and parallel to the SB plane, also observable in the oxidation activity. The knowledge of the structural–chemical changes provides a deeper understanding of the plastic deformation of metallic glasses especially for their functional applications and future improvements.

## Graphical abstract

Direct mapping of local atomic bonding and packing in deformed metallic glass is reported, with identification of the structural/chemical variation in and across the shear bands, forming the shear‐affected zones. The results confirm theoretical predictions for local softening in the deformed areas and provide necessary information to further engineer the catastrophic deformation failure of metallic glasses.

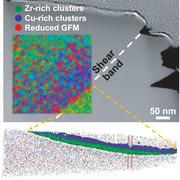

Metallic glasses (MGs) exhibit highly attractive properties^[^ ^1^, ^2^ ^]^ compared to their crystalline counterparts, in some cases, with the potential to outperform conventional metallic alloys.^[^ ^2^, ^3^, ^4^ ^]^ Unfortunately, MGs are brittle due to the formation of shear bands, which promotes catastrophic failure of the material.^[^ ^5^, ^6^ ^]^ Knowledge of the local atomic rearrangement (i.e., local structural descriptors: atomic distances, coordination, as well as chemical variations) induced by shear banding is quintessential to understand the deformation in MGs. Numerous efforts have been devoted to discover the underlying mechanisms, both experimentally and theoretically.^[^ ^7^, ^8^ ^]^ The nanometer sized width of shear bands (SBs) and their weak contrast to the surrounding matrix in most conventional characterization techniques^[^ ^9^, ^10^ ^]^ create difficulties in the detection and characterization of SBs. Recent methodological improvements have enabled some progress. For example, scanning transmission electron microscopy (STEM) high‐angle annular dark‐field (HAADF) imaging, nanobeam X‐ray fluorescence, and high‐energy X‐ray tomography showed a lower signal in SBs, created by uniaxial deformation, compared to the surrounding matrix. The observations associated the contrast to density reduction^[^ ^11^ ^]^ and cavities^[^ ^12^ ^]^ in the SB. STEM‐HAADF imaging and fluctuation electron microscopy (FEM)^[^ ^10^, ^13^, ^14^, ^15^, ^16^ ^]^ revealed variation of the intensity and local diffraction patterns along SBs in several types of multi‐axially deformed MGs. The variations were associated with density and structural fluctuations; atom probe tomography (APT)^[^ ^17^ ^]^ confirmed Al redistribution along SBs in a AlYFe MG, indicating that chemical modifications also occur during shear banding. Despite the aforementioned advances, experimental information on the direct atomic arrangements in the SBs is still largely missing. In contrast, molecular dynamics (MD) simulations offer an explicit picture of the structure in MGs, where the network of geometrically (energetically) favored motifs (GFMs) (polyhedral clusters)^[^ ^18^ ^]^ (e.g., full icosahedra in Cu‐rich CuZr MGs^[^ ^19^ ^]^) offers a stiff framework responsible for the high strength of glasses.^[^ ^20^ ^]^ MD predicts that during shear banding the GFMs and their network break down during SB initiation and are subsequently transformed to fragmented clusters.^[^ ^21^, ^22^ ^]^ However, no experimental confirmation for the polyhedral cluster order in SBs is available yet due to the difficulties of conventional diffraction and imaging techniques to characterize amorphous nanovolumes.

It has been realized that the nanoscale width of SBs is not enough to store the energy during plastic deformation of MGs.^[^ ^23^ ^]^ Local hardness,^[^ ^24^ ^]^ strain distribution,^[^ ^25^, ^26^ ^]^ and rearrangement of magnetic domains^[^ ^27^ ^]^ measured in deformed MGs indicate the existence of the so‐called shear band affected zones (SBAZ) extending up to micrometers from the SBs as a result of residual shear strain. A recent FEM study indicated that there is a structural difference between the vicinity of SBs and the undeformed matrix.^[^ ^14^, ^15^, ^16^ ^]^ Up to now, the explicit depiction of the atomic arrangement in the SBAZs, their nanoscale spatial distribution respect to the SBs and the structural–chemical correlation during plastic deformation are not yet known.

We utilized atomic pair distribution function (PDF) as a structure descriptor and combined it with 4D‐STEM to map the PDF locally at the nanoscale (STEM‐PDF),^[^ ^28^, ^29^ ^]^ followed by independent component analysis (ICA)^[^ ^30^ ^]^ to overcome the aforementioned challenges. By further correlation with APT results, we provide an explicit view of the SB and SBAZ, targeting the missing information of bonding, coordination environment, atomic packing geometry, and chemistry, visualizing their nanoscale distribution. We experimentally confirm, for the first time, the reduction of GFM polyhedra and their face share connections in SBs (which gives rise to a more liquid‐like nature), as well as the modification of local atomic chemical environment in SBs and SBAZs. Our observations portray that the energy stored during plastic deformation is allocated both in the SB and the SBAZs. We further revealed the microscopic origin of the significantly enhanced diffusion path in the SB compared to the SBAZs and bulk.

The structural and chemical analysis of SBs and SBAZs was conducted for the commercially available and widely studied Vitreloy 105 MG (Zr_52.5_Cu_17.9_Ni_14.6_Al_10_Ti_5_ at%).^[^ ^2^, ^3^, ^4^, ^5^, ^11^, ^12^, ^14^, ^31^ ^]^ A large number of SBs were obtained (illustrated in **Figure** **1**a) by deforming the material using high‐pressure torsion (HPT). The position of the SBs was identified by the shear steps on the material surface, and lift‐out TEM lamellae were prepared using focus Ion beam (FIB) as shown in Figure 1b,c. The STEM‐HAADF image (Figure 1d) shows shear steps on the sample surface below which the SBs are present. The relative intensity profiles (defined as $\Delta =\frac{I_{\text{SB}}-I_{M}}{I_{M}}$, where *I*_SB_ and *I*_M_ are the intensity of the SB and the matrix) perpendicular to the SBs are plotted in Figure 1e. Only extremely weak contrast of the SBs is observed in the STEM‐HAADF images, at the border of the detection limit, in contrast to previous reports.^[^ ^10^, ^11^, ^12^, ^13^ ^]^ In a later section, it will be shown that strong contrast is observed after exposing the SBs to oxygen. Nevertheless, the shear offsets at the free surface are clear indicators for shear banding and are used to locate the SBs in the following study.

**Figure 1:**
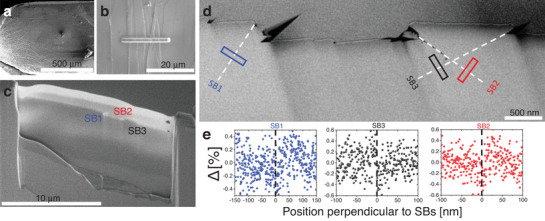
Overview of SBs in Vitreloy 105 after HPT showing the location of shear steps. a) SEM overview image showing areas with massive presence of shear bands. b) SEM image showing the area of interest that was used for FIB lift‐out. c) SEM image showing the lamella cross‐section and the area where SBs are located. d) STEM‐HAADF image showing SBs decorated with dash lines, labeled as SB1, SB2, and SB3. e) The relative intensity change for SBs 1–3 taken from the rectangular regions in (d), the dashed lines indicate the SB position, and the standard deviations (error bars of the intensity) are 0.24%, 0.22%, and 0.22% for SBs 1–3, respectively.

A 4D‐STEM map containing a single SB (SB1 in Figure 1e) was acquired at the area indicated by the dashed black box in **Figure** **2**a for local PDF analysis. PDF translates the information from the diffraction pattern to interatomic distances and atomic coordination, thus directly describing the short‐range order (SRO) and medium‐range order (MRO) configuration of the glassy structure.^[^ ^32^, ^33^ ^]^ STEM‐PDF, schematically shown in Figure S1, Supporting Information, utilizes the 4D‐STEM data to visualize the local structure information with nanometer spatial resolution.^[^ ^28^, ^29^ ^]^ The obtained STEM‐PDF array was analyzed using ICA, which was recently applied to STEM‐PDF for characterizing a variety of amorphous composites.^[^ ^34^, ^35^ ^]^ ICA was developed for “blind source separation” minimizing the mutual information between the estimated source signals using a statistical analysis of the data.^[^ ^30^ ^]^ The de‐mixed source signals are called independent components (ICs), representing statistically independent structural constituents in the MG. Hence, each PDF in the STEM‐PDF array can then be expressed as a linear combination of the ICs. ICA of the STEM‐PDF data cube reveals two ICs (Figure 2b, described in detail in Experimental Section) exhibiting distinct features. The first peak of the ICs represents the direct metallic bonding, that is, the nearest neighbors (NN) distances, while the second peak represents the second‐order NN correlations. As reported in the X‐ray study of CuZr^[^ ^36^ ^]^ and MD simulated NiZr glasses,^[^ ^37^ ^]^ the relative contribution of the elemental specific NN bonds affects the shape and apex position of the PDF peaks, making the PDF sensitive to structural changes caused by varying the elemental composition. Based on the average Cu—Cu, Cu—Zr, and Zr—Zr NN distances, determined by MD simulations of a Zr_50_Cu_50_ glass,^[^ ^38^, ^39^ ^]^ one can conclude that IC1 reflects the packing of atoms with larger distances and can be attributed to Zr—Zr correlations (indicating a Zr‐rich configuration). IC2 reflects the packing of smaller atoms and can be attributed to a Zr‐poor configuration (i.e., X—X and X—Zr, where X denotes Cu, Ni, Ti, and Al correlations).

**Figure 2:**
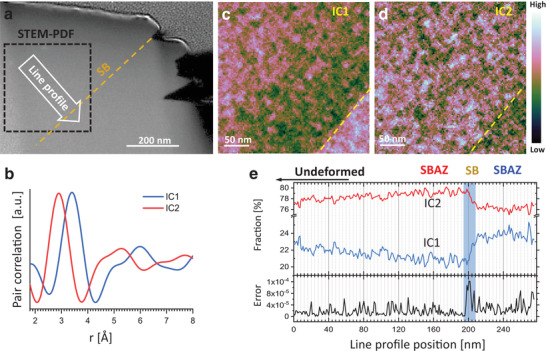
STEM‐PDF and ICA of a SB and its SBAZs. a) STEM‐HAADF image of the area located below the shear offset. b) ICs of the STEM‐PDF maps indicating the Zr—Zr (blue curve) and X–X, X–Zr (red curve) specified atomic pair correlations (X denotes Cu, Ni, Ti, Al). c,d) Spatial distribution maps of the two ICs. e) Line profile of the weighting coefficients of the ICs (top, colored lines) following the direction of the arrow in (a) perpendicularly to the SB. The misfit error (bottom, black line) indicates that the SB structure contains more components than IC1 and IC2. The shaded blue bar highlights the SB.

Figure 2c, d show the spatial distribution of IC1 and IC2. The maps clearly show that the glassy structure varies with a sharp interface (yellow dashed lines) at the location of the shear band, which can be hardly seen by conventional electron microscopy techniques (such as in Figure 1d,e). A line profile of weight fractions of ICs perpendicular to the SB is drawn in Figure 2e. It reveals that the concentration of the Zr‐rich structure (IC1, blue curve) gradually decreases from the matrix (top left of Figure 2c,d) toward the SB forming a Zr‐poor region, then drastically raises in the SB and remains at a high weight fraction on the other side of the SB, forming a Zr‐enriched region (bottom right in Figure 2c,d). IC2 exhibits the opposite behavior, the concentration of the Zr‐poor structure (IC2, red curve) slightly increases before the SB, steeply reduces in the SB, and remains low after the SB. These results clearly reveal an inhomogeneous distribution of IC1 and IC2 in the regions surrounding the SB, and are explicit structural evidence for the existence of two different SBAZs. The maximum structural inhomogeneity occurs in the SB, however, with a weight fraction change less than 3%. Hence, the variation of related elemental concentrations is expected much below 3% and difficult to observe with conventional techniques.

Figure 2e (bottom, black line) shows the fitting error of the two ICs to the PDFs. There is a significantly higher fitting error at the SB position. This indicates that the atomic structure in the SB is more complex than the description by the two ICs and additional structural motifs are present in the SB. Moreover, the error plot quantifies the width of the SB, from the atomic structure point of view, to be ≈12 nm. To inspect the structural variations in the SB, a line profile of local PDFs across the SB (position indicated by the white arrow in Figure 2a) is plotted in **Figure** **3**a. The temperature‐type color corresponds to the amplitude of the PDFs, while the horizontal and vertical axis represent the atomic distance *r* and the scanning positions on the sample. Shifting features are obvious (in particular at *r* ≈ 3.4 and ≈4.8 Å) in the SB region, which divides the PDF line‐scan into three regions: A Zr‐depleted SBAZ, the 12 nm wide SB, and a Zr‐rich SBAZ (as marked by the colored braces in Figure 3b), in line with the ICA results (Figure 2c–e). Averaged PDF of each of the three regions are plotted in Figure 3c for further visualization, where the black, red, and blue marks at 2.6, 2.8, and 3.2 Å indicate the average NN distances of Cu—Cu, Cu—Zr, and Zr—Zr determined by MD simulations of a Zr_50_Cu_50_ glass.^[^ ^38^, ^39^ ^]^ Therefore, the continuous shift of the feature (in the direction from top to bottom in Figure 3b) in region *r* = 3.20  to  3.70 Å in the SB is due to an increased Zr—Zr NN as reflected by the PDF amplitude (green line, right side Figure 3b). Interestingly, the shoulder of the second PDF peak at *r* = 4.77 Å, which corresponds to the second (X—X and X—Zr) coordination shell, does not exhibit a gradual change, but drops abruptly in the SB (orange line, right side Figure 3b). To understand the meaning of the shift of the second PDF peak, we consider the generally accepted consensus that the atoms in MGs are tetrahedrally close‐packed forming Kasper polyhedral motifs.^[^ ^18^, ^40^ ^]^ They can be grouped as GFM, for example, Cu‐centered icosahedra in Cu‐rich CuZr MGs, and geometrically unfavored motifs (GUM). The connection schemes of the tetrahedra can be described as vertex (VS, 1‐common‐atom), edge (ES, 2‐common‐atom) and face (FS, 3‐common‐atom) sharing (examples in Figure S2a,b, Supporting Information).^[^ ^41^, ^42^ ^]^ The different sharing types lead to different distances of the second NNs in the glass (2*R*_NN_ for VS, $\sqrt{3}R_{\text{NN}}$ for ES, and $\sqrt{\frac{8}{3}}R_{\text{NN}}$ for FS, where *R*_NN_ is the average NN distance). Typically, a mixture of all types is present, giving rise to a broad second peak in the PDF^[^ ^39^, ^41^, ^42^ ^]^ (Figure S2c–f, Supporting Information). The FS tetrahedra provide the most efficient, dense atomic packing and are the building blocks for constructing the GFMs. FS tetrahedra are also responsible for the formation of a percolated interconnected GFM network, which acts as the backbone of the MG and provides the high stiffness and thermal stability. Therefore, the population of FS tetrahedra has been recognized to play a central role in the deformation of MGs.^[^ ^19^, ^39^, ^41^, ^42^, ^43^ ^]^ The above‐mentioned PDF peak at ≈4.8 Å corresponds to the FS tetrahedral packing and the “abrupt” reduction of this peak at the SB (orange line, right side Figure 3b) indicates a sudden decrease of FS tetrahedra. The population of the FS tetrahedra is visualized in Figure 3d by plotting the spatial distribution of the PDF amplitude at $r=\sqrt{\frac{8}{3}}R_{\text{NN}}$ (where *R*_NN_ was determined by finding the mass center of the first peak in each PDF). A weak but visible dark line corresponding to reduced density of FS tetrahedra along the position of the shear band can be observed (guided by golden arrows in Figure 3d). Meanwhile, the averaged PDFs taken from the shear band (Figure 3c) also show a clear reduction of FS tetrahedra (reduced intensity of the PDF at ≈4.8 Å) in the SB compared to the surrounding material, which further confirms the contrast in the FS tetrahedra map (Figure 3d). The results indicate the fragmentation of GFMs in the SB. This observation provides a direct experimental proof for the theoretical expectation,^[^ ^22^ ^]^ that GFMs convert to fragmented polyhedra (i.e., GUMs) to enable plastic flow in SBs. This also explains the assumption of enhanced “free volume” in SBs.

**Figure 3:**
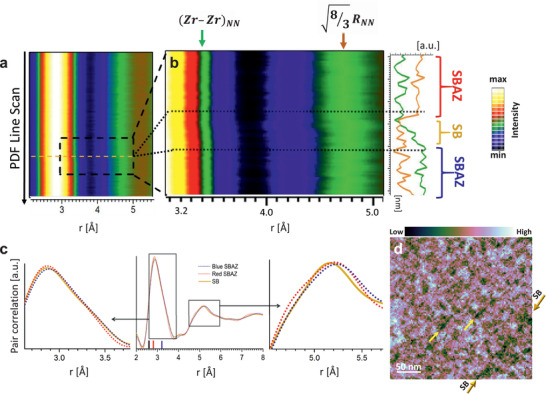
Detailed PDF analysis and map of the face‐shared tetrahedra. a) PDF line scan following the direction of the arrow in Figure 2a perpendicularly to the shear band. The horizontal axis represents the atomic pair distance *r* (Å) and the vertical axis corresponds to the scan position on the probed material. The temperature‐type color corresponds to the amplitude of the PDFs, that is, the population of pair correlation. b) Enlarged part of (a) inspecting the structure transition across the SB. The line chart at the right is the intensity line profiles vertically along the r positions highlighted by the green and brown arrows at 3.4 and 4.8 Å, respectively, indicating the feature influenced by the Zr—Zr NN and face‐shared polyhedral MRO, where the space between the minor marks in the vertical axis is 1 nm. c) PDFs of the SB (golden solid line), Zr‐depleted (red dotted line), and Zr‐rich (blue dotted line) SBAZs calculated from the averaged diffraction patterns of the areas marked by the golden, red and blue half‐parenthesis in (b). Reference marks at 2.6, 2.8, and 3.2 Å correspond to the Cu—Cu, Cu—Zr, and Zr—Zr NN length in Zr_50_Cu_50_ glass estimated by MD simulation.^[^ ^38^, ^39^ ^]^ d) Map of face‐share (FS) connected tetrahedral MRO by taking the intensity of PDFs of the STEM‐PDF map at $r=\sqrt{\frac{8}{3}}R_{\text{NN}}$, *R*_NN_ is the averaged first peak position of each PDF. The golden arrows outside the image are to indicate the shear band location. The two yellow arrows highlight visible local GFM depletion aligned in the shearing direction.

STEM‐PDF analysis is also performed in an area where two SB planes are intersected and cracks/shear offset are present (Figure S3a–b, Supporting Information). The IC maps (Figure S3c,d, Supporting Information) indicate the location of the SBs matching the shear offset and cracks. The PDFs taken from the SB area and SBAZs show the same behavior as the above analyzed SB and its SBAZs (Figure S3e, Supporting Information). Noticeable, the Zr‐rich SBAZ is constrained to a corner formed by the SB intersection, where the largest shear strain and material flow during deformation is expected.

3D APT reconstruction (**Figure** **4**a) of the deformed region in Vitreloy 105 clearly shows Cu‐ and Zr‐rich zones along the SB, while the undeformed bulk shows no significant chemical fluctuation (Figure 4b,d). The concentration profile (Figure 4c), extracted from the cylinder in Figure 4a (perpendicular to the SB plane as schematically shown in Figure 4e), exhibits a high asymmetry perpendicular to the SB, that is, on one side the SB is rich in Cu ≈23 at% (depleted in Zr) and on the other side the SB is rich in Zr ≈57 at% (depleted in Cu), while the concentration of the other elements remains unchanged. An obvious elemental fluctuation occurs within ≈12 nm, which agrees with the SB width observed by STEM‐PDF (Figures 2 and 3). To analyze the atomic distributions in the immediate vicinity around each atom in the SB and SBAZs, a fifth nearest neighbor (5NN correlation shell) distribution analysis was performed on the experimental APT data and compared to the randomized distribution of the atoms in the bulk.^[^ ^44^ ^]^ The investigation is restricted to the inter‐atomic distances of the 5NN to enhance the statistics and is displayed in **Figure** **5**. The bulk is well represented by the random model, however, obvious deviations are observed between the SB/SBAZ and the bulk (Figure 5a,b). The peak position reflects the elemental‐specific atomic density in the vicinity of an atom and a peak shift to smaller distances correspond to clustering of Zr and Cu atoms as seen in Figure 5a,b. The red SBAZ shows a higher degree of Cu clustering compared to the blue SBAZ, which shows a higher degree of Zr clustering; the SB shows the highest degree of elemental segregation. These results are in agreement with the STEM‐PDF findings. Moreover, random Ni—Ni, Al—Al, and Ti—Ti 5NN distributions in the bulk, SBAZs, and SB (Figure 5c–e) confirm that Ni, Ti, and Al have not segregated. Hence, the observed variation in the STEM‐PDF can be attributed mainly to variations of Cu—Cu, Cu—Zr, and Zr—Zr pairs, and IC2 can be related to Cu—Cu and Cu—Zr correlations.

**Figure 4:**
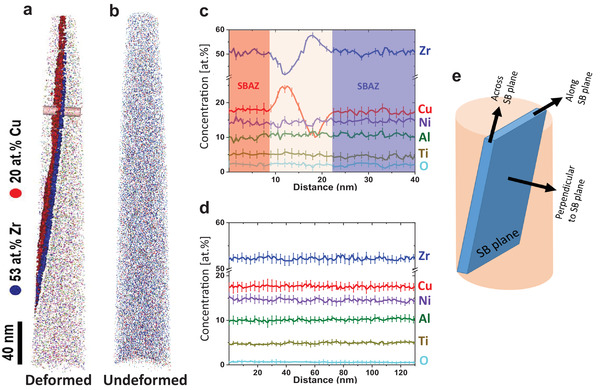
APT analysis of the deformed and undeformed bulk Vitreloy 105 (Zr_52.5_Cu_17.9_Ni_14.6_Al_10_Ti_5_). a) 3D reconstruction of the deformed sample containing a SB. Transport of Cu and Zr across the SB is obvious in the elemental map, being demarcated using Cu (red: 20 at%) and Zr (blue: 53 at%) isosurfaces along SB. b) The 3D elemental map, and individual elemental distributions for the undeformed bulk. c) 1D composition profile perpendicular to the SB taken from the cylinder in (a). d) Concentration–depth profile of all elements in the undeformed bulk. e) SB plane schematic showing the direction for concentration profiles analysis in APT.

**Figure 5:**
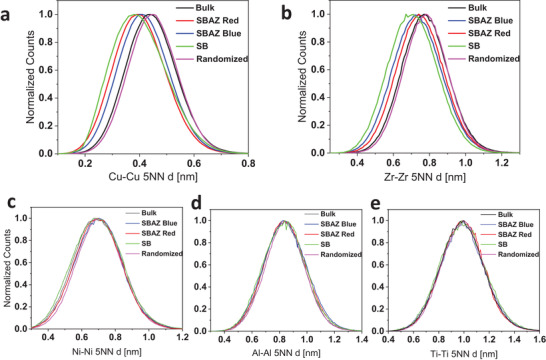
Fifth nearest‐neighbor (5th‐NN) distribution analysis for bulk, SBAZs, and SB obtained from deformed region and undeformed bulk areas. a) Cu—Cu 5th‐NN pairs. b) Zr—Zr 5th‐NN pairs. c) Ni—Ni 5th‐NN pairs. d) Al—Al 5th‐NN pairs. e) Ti—Ti 5th‐NN pairs. Experimental distributions are presented with corresponding random comparator.

Our STEM‐HAADF studies on freshly prepared FIB lamellas (with many shear steps) showed hardly any detectable contrast for the SBs in a variety of samples with different thickness, even though great efforts to align the SBs parallel to the electron beam have been made. In contrast, a lamella stored in a glove box (O_2_ <0.05 ppm) for 20 days (**Figure** **6**a) showed clear alternating SB contrast (Δ = −0.5%, in the darker SB area, Figure S4a, Supporting Information) similar to previously reported observations.^[^ ^13^, ^14^ ^]^ This is an indication for a structure prone to oxidation and differs from the SBAZs. The sample was further thinned (≈30 nm) and immediately examined by STEM‐HAADF, revealing that the alternating contrast in the SB disappeared (Figure 6b). The contrast in the SB was regained after 10 more days of storage in the glove box, and further sample thinning (≈30 nm) resulted in the SB not being visible again (Figure S4c–e, Supporting Information). Thus, the observed contrast seems to arise from preferred oxidation within the SB. Therefore, an APT tip containing a SB was prepared and stored in air for 3 days to investigate the oxidation effect. The oxygen heat map (Figure 6c and the corresponding 3D reconstruction in Figure S4f, Supporting Information) and the chemical analysis perpendicular to the SB (Figure 6d) reveal a drastic enrichment of oxygen in the SB up to 24 at%, while the amount of Zr and Ti decreases to 28 and 1.5 at%. In contrast, the oxygen content outside the SB is 1–2 at%. These results hint toward the formation of Zr oxide, specifically in the Cu‐rich side of the SB (the concentration profile including the Zr oxide is plotted in Figure S4g, Supporting Information). According to the oxygen contour across the SB plane (Figure 6e), oxygen is diffusing from the surface to the middle of the tip through the SB plane. This can be attributed to an interface enhanced corrosion, whereas the bulk and SBAZs without abrupt structural–chemical variations are passive.^[^ ^45^ ^]^ Radio tracer diffusion revealed an unexpected high diffusion coefficient along SBs, close to that of liquid metals, which was attributed to a possible interfacial diffusion along SBs and to inhomogeneous distribution of “free‐volume” perpendicular to the SB plane.^[^ ^46^ ^]^ We observed a clear reduction of GFM and their FS connection in the SB (Figure 3), suggesting a more “liquid‐like” behavior.^[^ ^19^, ^22^, ^39^ ^]^ Furthermore, Figure 6f shows fluctuation of oxygen concentration along the SB suggesting that there is a structure–chemical variation in the oxidized SB, which most probably resulted from the Eshelby‐type force,^[^ ^47^ ^]^ and seems to be strongest in the Cu‐rich region. Our results explain that the alternating contrast observed by STEM‐HAADF of the stored sample (Figure 6a) is enhanced by localized oxidation in the SB (even at the low oxygen levels in a glove box), and its disappearance after FIB thinning depends on the oxygen diffusivity across the SB plane.

**Figure 6:**
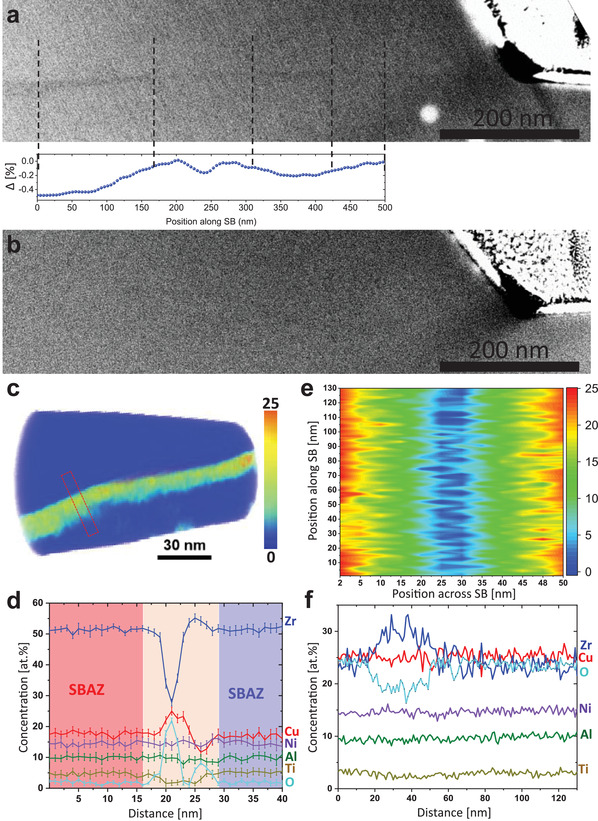
SB oxidation observation by STEM‐HAADF and APT. a) STEM HAADF image of a SB after 20 days sample storing. The plot shows the alternating contrast along the SB. b) STEM‐HAADF image obtained after FIB milling revealing that the SB contrast observed in (a) is not visible. c) Heat map showing the oxygen distribution in the deformed sample along the SB, the color bar is the oxygen concentration (at% units). d) 1D concentration profile perpendicular to the SB obtained from the region of interest drawn in (c). e) Contour composition oxygen profile showing the oxygen diffusivity from the surface through and across the SB plane in (c), the color bar is the oxygen concentration (at% units). f) 1D composition profile showing oxygen fluctuation along the SB in (c). Refer to Figure 4e for the schematic representation of extracted chemical concentration profiles in APT analysis.

In many experimental studies,^[^ ^5^ ^]^ it is proposed that during shear deformation regions with more free volume start deforming along the shear direction, localizing strain and triggering the neighboring clusters to undergo plastic deformation. MD studies indicate that GUMs contain more excess volume, potentially relating to the concept of “free volume,”^[^ ^48^ ^]^ and are more susceptible to be involved in shear transformation zones under continuous strain.^[^ ^42^, ^49^, ^50^ ^]^ Our experimental observation confirms the GFM to GUM conversion in the shear band and bridges the experimental and theoretical concepts. Further fragmentation of GFMs into GUMs eventually combines the enhanced free volume and forms the shear band.^[^ ^22^ ^]^ According to MD simulations, the GUMs will prefer different elemental composition than the GFM.^[^ ^19^, ^51^ ^]^ Hence, during shear band formation local chemical rearrangements take place and relax the structure in the SB. Among the major elements (Zr, Cu, Ni, Al) in Vitreloy 105, Cu has the highest mixing enthalpies, being the most “immiscible” element (the negative mixing enthalpy of unlike atoms stabilizes glass structures).^[^ ^52^ ^]^ As a consequence, formation of GUMs raises the energy of the system, which is then balanced by Cu segregation in the shear band. This is corroborated by the observed local enrichment of elemental‐specific bonds and chemical variation in our results. The rearrangement of Cu atoms will render more flexibility of the local chemical modification in the shear band and reduce the energetic barrier to convert the GFMs to GUMs promoting the shear band formation. This also explains the observed elemental variation in plastically deformed Al_85.6_Y_7.5_Fe_5.8_^[^ ^17^ ^]^ MG and Fe_25_Tb_75_^[^ ^53^ ^]^ nanoglass. It is reasonable to expect that by increasing the GUM to GFM ratio, for example by pre‐deformation, one can improve the ductility of the MG. It has been demonstrated by several studies that pre‐loading MGs (i.e., cold rolling, compression, ball milling, HPT, etc.) results in enhanced ductility.^[^ ^54^, ^55^, ^56^ ^]^ Meanwhile, modulating the energetic barrier to convert GFMs to GUMs or raising the cost for structure changing in GUMs under deformation may also suppress strain localization and results in increased toughness. This could be achieved by selecting atomic species that have limited flexibility to rearrange their local chemical structure, for example, a recent MD study^[^ ^57^ ^]^ shows that the slow kinetics of the Pd diffusion in Pd_82_Si_18_ glass plays the major role to prevent cavitation, enabling more extensive plastic strains before fracture in comparison to the CuZr system.

Furthermore, our observation reveals the structural/chemical modification spanning over hundred nanometers from the SB (one rich in Zr—Zr bonds and the other in Cu—Cu/Zr bonds, Figures 2 and 3) indicating the existence of SBAZs in the atomic level. This may explain the tens of micrometers asymmetric strain field perpendicular to the SB plane reported by magnetic domain analysis^[^ ^27^ ^]^ and scanning X‐ray diffraction.^[^ ^26^ ^]^ The curved surface at the SB offset (**Figure** **7**a, blue bracket) indicates that weak plastic flow occurs in the upper SBAZ, where the material suffered tensile strain, during shearing.^[^ ^21^, ^25^ ^]^ By extrapolating the curving point parallel to the SB (white dashed lines in Figure 7a), one can see a good coincidence with the lateral length of the SBAZ. In fact, we did observe local GFM depletion aligned in the shearing direction (yellow arrows in Figure 3d) and increase of Cu‐rich clusters (Figures 2 and 5) in the Zr‐depleted SBAZ. One might speculate that the Cu‐rich clusters increase the population of the Cu‐centered icosahedra and enhances the stiffness^[^ ^40^ ^]^ preventing further flow and contributes to the localization of plastic deformation mainly in the SB. On the other side of the SB, the Zr depletion leads to enhanced ductility in the lower SBAZ, favoring multiple SB formation^[^ ^5^ ^]^ as shown in Figure 7a. Moreover, the elemental clustering in the SBAZ and SB reduces the chemical SRO and increases the enthalpy of the system, hence the applied energy during plastic deformation is stored not only in the SB but also in the SBAZs.

**Figure 7:**
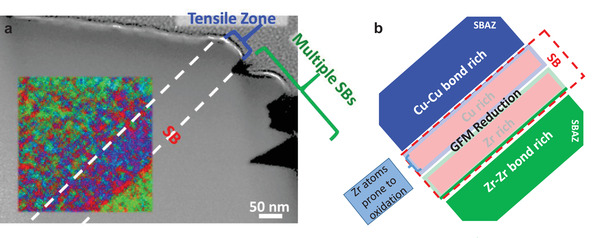
SB and SBAZs summary. a) Superposition of the IC maps and GFM map with the STEM‐HAADF image of the deformed area in SB1, where green represents the fraction of IC1, blue IC2, and red corresponds to GFM reduction. b) Representation of the different glass structures found in this study.

The local chemical–structural modifications associated to shear banding are unveiled, summarized in Figure 7. A reduction of GFMs (confined to a few nanometers forming the SB) and abrupt segregation of Zr and Cu at opposite sides inside the SB are observed. The Zr atoms in the Cu rich section of the SB are prone to oxidize, giving rise to alternating contrast along the SB. In the vicinity of the SB, Cu, and Zr tend to invoke clustering, extending several hundred nanometers away from the SB, differing slightly in bond type (Cu—Cu and Zr—Zr bonds enrichment) and chemical composition compared to the undeformed matrix, forming the antisymmetric SBAZs. The findings present an explicit view of the changes of the atomic bonding and packing environment in the SBAZs and SBs induced by plastic deformation.

## Experimental Section

Vitreloy 105 (Zr_52.5_Cu_17.9_Ni_14.6_Al_10_Ti_5_ at%) disc with a diameter of 15 mm was electro‐discharge machined from a Vitreloy 105 MG (supplied by Liquidmetal Lake Forest, California) rectangular plate (27 mm × 75 mm × 0.75 mm). The disk was cut into two halves and the internal surfaces of both halves were polished with a series of diamond suspensions (9, 3, and 1 µm) followed by 50 nm colloidal silica. Subsequently, the half disks were joined together with a thin layer of a lacquer (in order to prevent full adhesion of the two parts) and processed by quasi‐constrained HPT at room temperature under applied pressure of 6 GPa for ¼ revolution using anvils with grooves of 0.25 mm, following a procedure similar to the one described by Gunderov et al.^[^ ^58^ ^]^ The disk thickness after processing was ≈0.65 mm. Figure 1a is a disk cross‐section representative image that shows shear bands as well as the area from which samples have been extracted for transmission electron microscopy (TEM) and APT characterization. The lamella extraction was performed by employing both FEI Strata 400 and Zeiss Auriga 60 focused ion beam systems for TEM and APT, respectively. Figure 1b is a representative zoomed out image of the areas selected for TEM and APT sample preparation, a platinum layer that is usually deposited over the area of interest to protect the sample from gallium ion beam damage and degradation can be seen in Figure 1b. The conventional STEM HAADF measurements were performed in nanoprobe mode with a camera length of 195 mm, 15 mrad semi convergence angle and spot size 5, resulting in ≈0.5 nm probe size.

The 4D‐STEM experiments for STEM‐PDF analysis were performed using an image‐Cs corrected Titan 80‐300 (FEI) operated at 300 kV in microprobe STEM mode with spot size 7, gun lens 6, extraction voltage of 4.5 kV and a 20 µm C2 aperture resulting in a semi convergence angle of 0.6 mrad. A camera length of 130 mm and a Medipix3 pixelated detector (Quantum Detector Ltd.) was used for the data acquisition. These settings result in a probe size of ≈1.7 nm. The largest recorded diffraction angle is 2θ_max_ = 75 mrad (*s* = 2θ/λ = 2.5 Å^–1^), where θ is the scattering semi‐angle and λ is the wavelength of the incident electrons. The STEM‐PDF procedure is depicted in the schematic in Figure S1, Supporting Information. The 4D‐STEM acquisition (sketched in Figure S1a, Supporting Information) was obtained by laterally scanning the electron probe over the region of interest in a 256 by 256 scanning pixels matrix (65 536 diffraction patterns) per map, and the diffraction patterns were recorded on a Medipix3 detector (Merlin, Quantum Detector Ltd with frame size of 256 × 256 pixels) with an exposure time of 6 µs per diffraction pattern and a step size of 1.18 nm. To derive the PDFs from the 4D‐STEM data (Figure S1b, Supporting Information), the 2D diffraction patterns are azimuthally integrated to 1D diffraction profiles *I*(*s*), then the diffraction profiles are normalized to $\varphi \left(s\right)=\;\frac{I\left(s\right)-Nf{\left(s\right)}^{2}}{Nf{\left(s\right)}^{2}}s$ following the procedure described in literature,^[^ ^32^ ^]^ *f*(*s*) is the averaged atomic scattering factor of all elements contributing to the diffraction pattern, and *N* is the number of atoms irradiated by the electron beam. A correction of 〈ϕ(*s*)〉 by subtracting a fourth‐order polynomial function is performed after normalization. This can reduce the low frequency artifact in the local PDFs resulting from multiple scattering of electrons.^[^ ^33^ ^]^ As demonstrated in ref. ^[^ ^28^ ^]^ this also eliminates the issue that the local scattering factor may deviate from the average due to compositional fluctuations. The PDFs are then obtained by Fourier sine transformation of ϕ(*s*), that is, $\text{PDF}(r)=\int_{0}^{s_{\max}}\varphi (s)\sin(2\pi sr)ds$. The array of PDFs were arranged to form a PDF data cube following the scan sequence of the diffraction patterns in the 4D‐STEM acquisition. For a reasonable signal to noise ratio, the Fourier transformation uses *s*_max_ = 1.2 Å^−1^ for all the PDFs in the PDF data cubes (maps), and *s*_max_ = 2.5 Å^−1^ for the averaged PDFs in Figure 3 to improve the resolution of PDFs in *r*‐space.

To analyze the structural motifs in the deformed region including the shear band, principal component analysis (PCA) was applied followed by ICA to the STEM‐PDF data to obtain the structural information of individual phases affected by the shear banding. For increasing the signal to noise ratio in the PCA/ICA process, the PDF map is integrated parallel to the shear band direction (dashed golden line in Figure 2a,c,d). This results in a PDF line scan as shown in Figure 3a,b, the temperature‐type color corresponds to the amplitude of the PDFs (arbitrary unit). This line‐scan is input to the PCA/ICA using the FastICA code.^[^ ^30^ ^]^ PCA processes the data as a 2D matrix (*X*), where the rows are the PDFs as function of the atomic pair distance (in units of Å in this paper) and the columns correspond to different sample locations. The experimentally measured PDFs are considered as a linear combination of PDFs from the individual structural components. PCA diagonalizes the covariation matrix *X* ^T^ *X*, where *X* ^T^ is the transpose of *X*. The eigenvectors represent the component PDFs in the data and the eigenvalues describe the statistical significance of each component. Figure S5a, Supporting Information shows the first four components yielded from PCA. The elbow in the scree plot (Figure S5b, Supporting Information) suggests the first two components are the most principal. The two principal components selected by PCA are further processed by ICA to ensure their statistical independence, that is, minimized information overlap between each other. The similarity between the ICs shown in Figure 2b and the PCA results (Figure S5a, Supporting Information) shows that the principal components from PCA are close to be statistically independent. The IC maps shown in Figure 2c,d are then obtained by multiple linear least square fitting the two ICs to the STEM‐PDF data cube.

3D APT was performed on sharp tip shape samples, prepared using a Zeiss Auriga 60 FIB system. Prior to the lift‐out, a platinum protective layer (150 nm in thickness) was deposited over the area of interest to protect the APT sample from the gallium (Ga) ion beam milling damage. To produce the required atom probe specimen geometry, annular milling was used to create needle‐shaped morphology with a tip diameter smaller than 100 nm. The APT measurements were carried out using a Cameca‐LEAP 4000X HR instrument in laser pulse mode (wavelength 355 nm, pulse frequency 100 kHz, pulse energy 30 pJ, evaporation rate 0.50%) at 50 K. Data processing was realized with the CAMECA integrated visualization and analysis software (IVAS‐version 3.6.1), incorporating standard reconstruction algorithms that make it possible to extract the 3D nanoscale chemical distribution of all detected atoms in the analysis volume.

## Conflict of Interest

The authors declare no conflict of interest.

## Supporting information

Supporting Information

## Data Availability

Data available on request from the authors. Data available in the article's Supporting Information.
