# Supplementary material for: Unveiling the Local Atomic Arrangements in the Shear Band Regions of Metallic Glass
Source: Adv Mater. 2021 Feb 19;33(12):2007267. doi: 10.1002/adma.202007267 (PMC11468532; doi:10.1002/adma.202007267)
Supplement: Supplementary file 1 — Supporting Information [file ADMA-33-2007267-s001.pdf]

© 2021 Wiley-VCH GmbH

ADVANCED MATERIALS

Supporting Information

for *Adv. Mater.,* DOI: 10.1002/adma.202007267

Unveiling the Local Atomic Arrangements in the Shear Band Regions of Metallic Glass

Xiaoke Mu, Mohammed Reda Chellali,* Evgeniy Boltynjuk, Dmitry Gunderov, Ruslan Z. Valiev, Horst Hahn, Christian Kübel, Yulia Ivanisenko, and Leonardo Velasco**

Supporting Information

Unveiling the Local Atomic Arrangements in the Shear Band Regions of Metallic Glass

Xiaoke Mu, Mohammed Chellali* (equal contribution), Evgeniy Boltynjuk, Dmitry Gunderov, Ruslan Valiev, Horst Hahn, Christian Kübel, Julia Ivanisenko, Leonardo Velasco**

Dr. X. Mu, Dr. M. R. Chellali, Dr. E. Boltynjuk, Prof. H. Hahn, Dr. C. Kübel, Dr. Julia Ivanisenko, Dr. L. Velasco
Institute of Nanotechnology
Karlsruhe Institute of Technology
Eggenstein-Leopoldshafen, 76344, Germany
E-mail: xiaoke.mu@kit.edu
E-mail: mohammed.chellali@kit.edu
E-mail: leonardo.estrada@kit.edu

Dr. E. Boltynjuk, Dr. R. Z. Valiev
Saint Petersburg State University
St. Petersburg, 199034, Russia

Dr. D. V. Gunderov
Institute of Molecule and Crystal Physics
Ufa Federal Research Center RAS
Ufa, 450075, Russia

Dr. R. Z. Valiev
Ufa State Aviation Technical University
Ufa, 450008, Russia

Prof. H. Hahn, Dr. C. Kübel
Joint Research Laboratory Nanomaterials
Technische Universität Darmstadt
64206, Darmstadt, Germany

Dr. C. Kübel
Karlsruhe Nano Micro Facility
Karlsruhe Institute of Technology
Eggenstein-Leopoldshafen, 76344, Germany

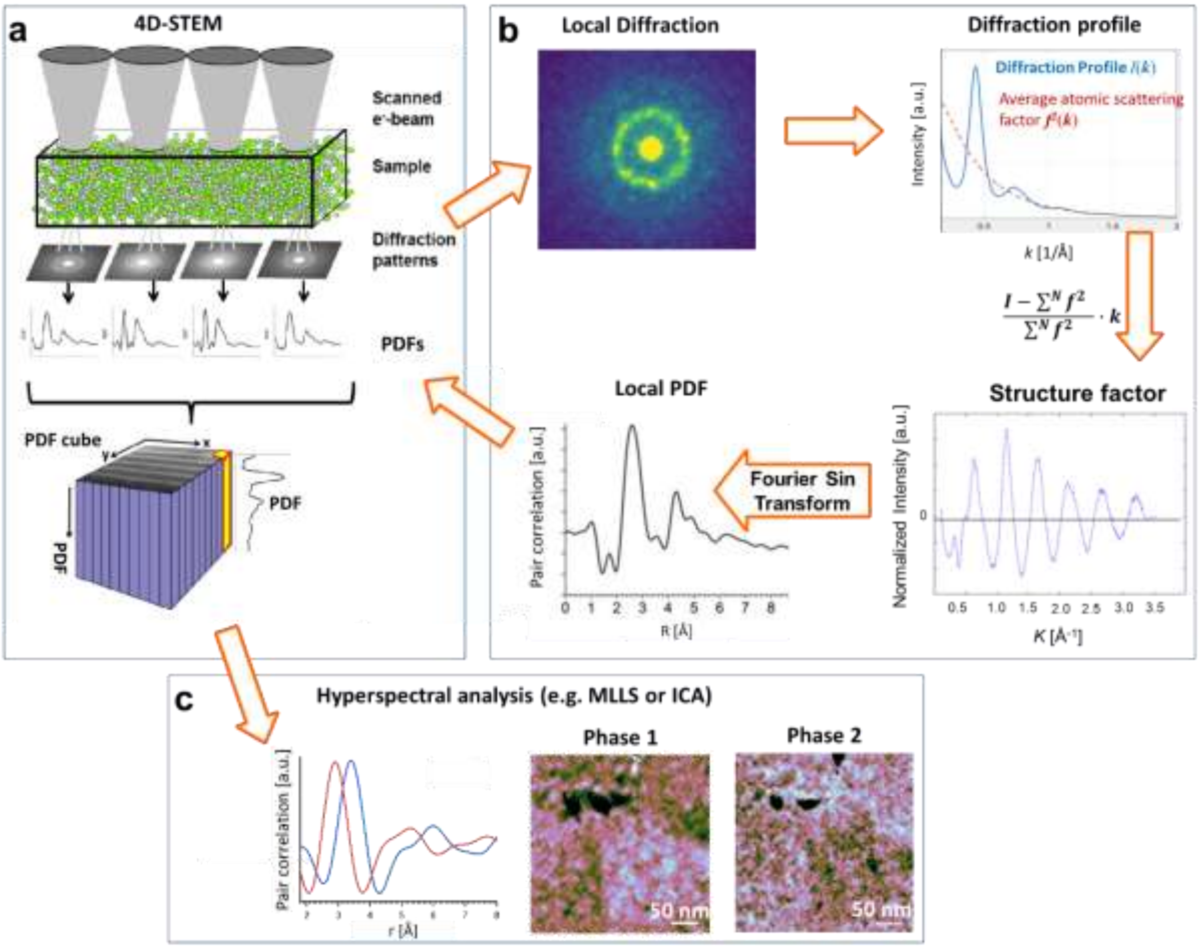

Figure S1. Work flow for the STEM pair distribution function (STEM-PDF). **a**, 4D-STEM acquisition and PDF cube creation. **b**, details of PDF calculation from individual local nanobeam electron diffraction. **c**, Hyperspectral analysis of the PDF data cube to obtain the structural map and PDFs for individual structural phases.

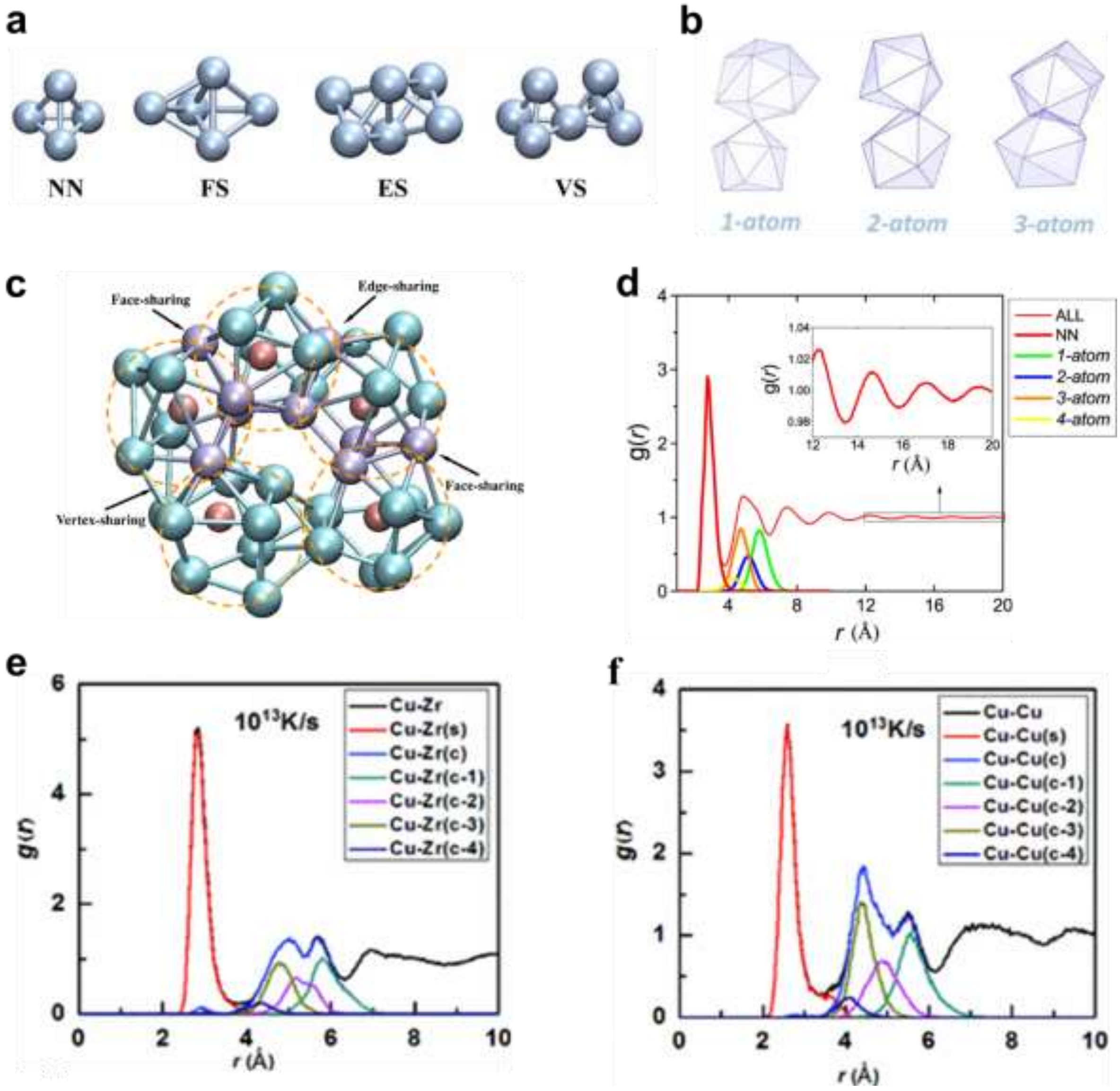

Figure S2. Connection Schemes for the tetrahedral and their influence on the PDFs in metallic glasses. Taken from literature for the convenience of readers. **a,** sharing schemes of tetrahedra in MGs[1], NN denotes nearest neighbor, FS is face share, ES is edge share and VS is vertex share. **b**, Connection schemes of polyhedral motifs[2]. **c,** interconnected polyhedra through the connection of tetrahedra illustrated in **a**[1]. **d**, PDF for $Zr_{46}Cu_{46}Al_8$ MGs obtained by MD simulation[2]. **e** and **f,** partial Cu–Zr and Cu–Cu PDFs for $Cu_{50}Z_{50}$ MG obtained by MD simulation[3] showing that the second peak of PDFs strongly reflects the polyhedra connection. Figure S2a and S2c Reprinted from [W. K. Luo, H. W. Sheng, E. Ma, *Appl. Phys. Lett.* **2006**, *89*, 131927], with the permission of AIP Publishing. Figure S2b and S2d Reprinted from [J. Ding, E. Ma, M. Asta, R. O. Ritchie, *Sci. Rep.* **2015**, *5*, 1.], (CC BY 4.0). Figure S2e and S2f reprinted from [S. P. Pan, J. Y. Qin, W. M. Wang, T. K. Gu, *Phys. Rev. B - Condens. Matter Mater. Phys.* **2011**, *84*, 092201], with permission of APS.

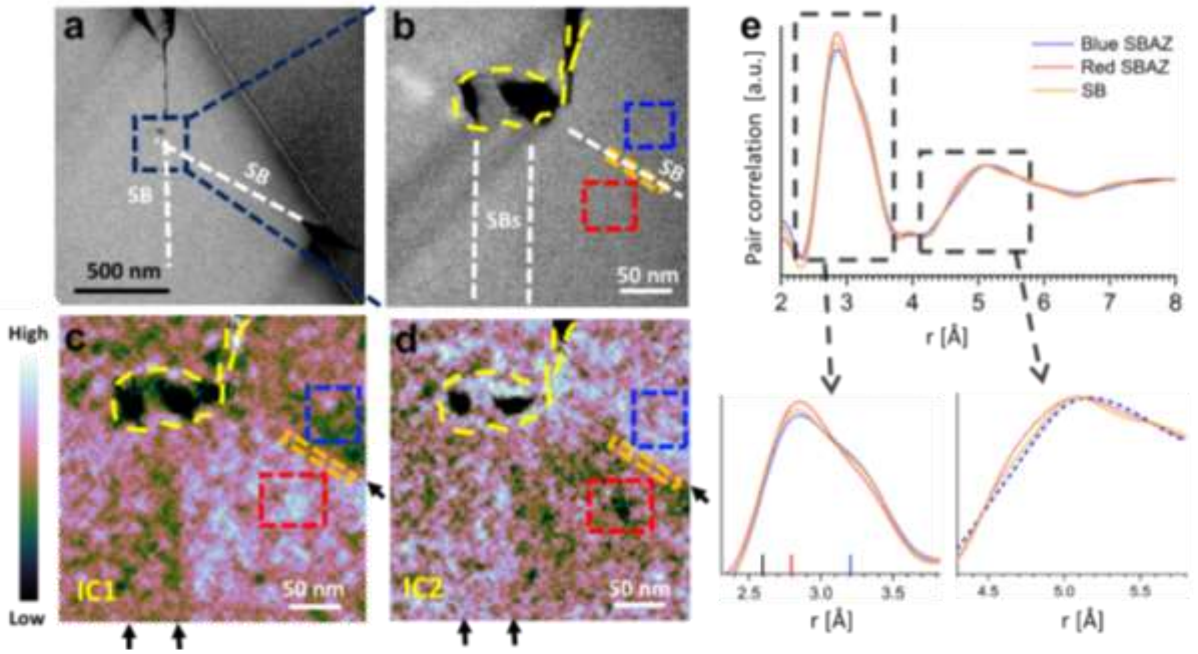

Figure S3. STEM-PDF analysis conducted in the intersection of two SBs. **a** and **b**, STEM-HAADF images of intersected SBs. The crack is circled by the yellow dashed curve. **c** and **d**, Distribution map of the IC1 and IC2, SB (black arrows) and two type of SBAZs can be observed. **e**, PDFs taken from the SB and SBAZs highlighted by the orange, red and blue dashed boxes in **b-d**. Reference marks at 2.6, 2.8 and 3.2 Å correspond to the Cu–Cu, Cu–Zr and Zr–Zr NN distance in $Zr_{50}Cu_{50}$ glass[3,4].

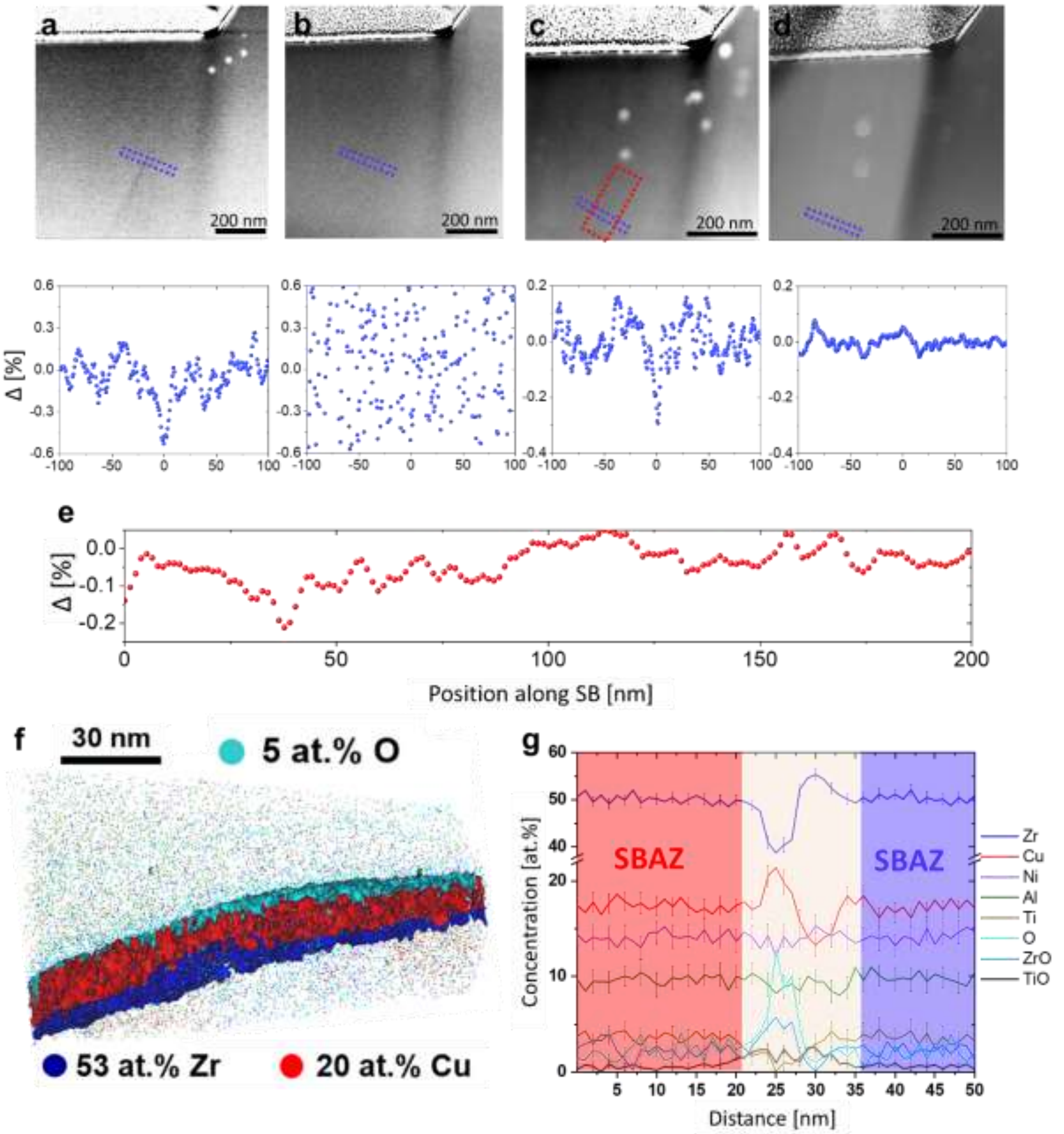

Figure S4. Shear band oxidation observation with STEM-HAADF and APT. **a-d**, STEM HAADF image of a SB in the same sample. **a**, after 20 days sample storing. **b**, after FIB thinning of **a**. **c**, 10 days after **b**, the SB alternating contrast is visible. **d**, FIB re-thinning of **c**. The relative intensity change profile standard deviations (error bar of intensity) are 0.14%, 0.55%, 0.08%, 0.02% for a-d, respectively. **e**, contrast profile extracted from the SB inside the red rectangle in **c.** **f**, 3D atom probe reconstruction of oxidized deformed sample including Oxygen, Cu and Zr depicted as aqua, red and blue isoconcentration surfaces, respectively. **g,** one-dimensional concentration profile perpendicular to the SB. Refer to Figure 4e for the schematic representation of extracted chemical concentration profiles in APT analysis

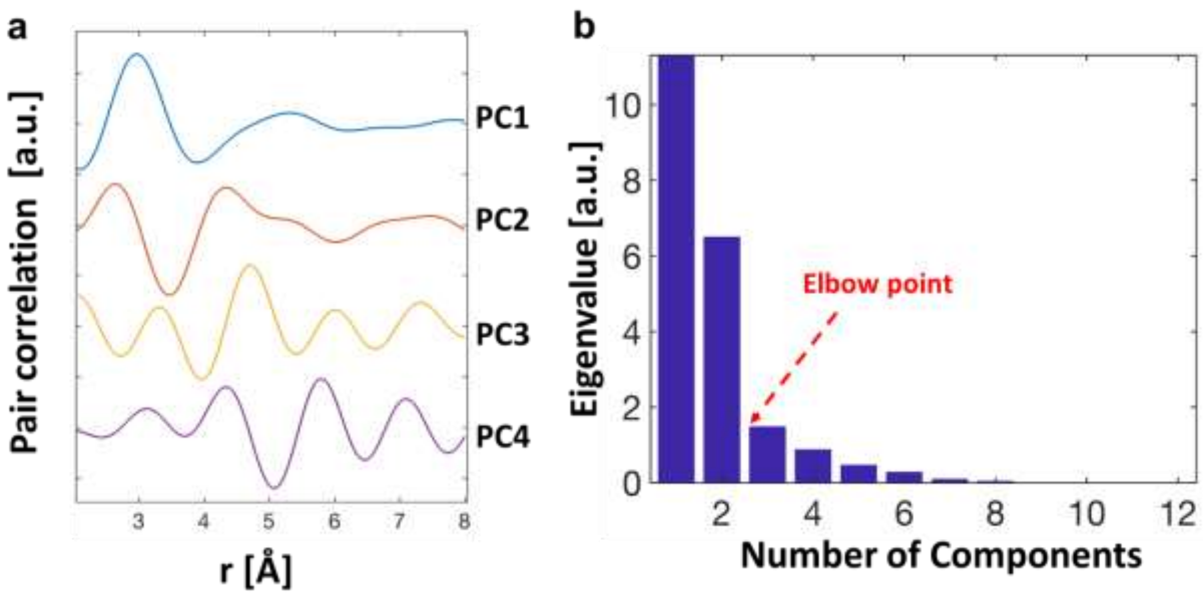

Figure S5. PCA analysis of the STEM-PDF data. **a**, the first four component PDFs in the order from top to bottom (PC is principal component). **b**, Eigenvalue scree plot for each component. The abrupt truncation (elbow point) after the 2nd component suggests the first two components should be considered as principal components.

References

[1] W. K. Luo, H. W. Sheng, E. Ma, *Appl. Phys. Lett.* **2006**, *89*, 131927.

[2] J. Ding, E. Ma, M. Asta, R. O. Ritchie, *Sci. Rep.* **2015**, *5*, 17429.

[3] S. P. Pan, J. Y. Qin, W. M. Wang, T. K. Gu, *Phys. Rev. B - Condens. Matter Mater. Phys.* **2011**, *84*, 092201.

[4] S. Marinier, L. J. Lewis, *Phys. Rev. B - Condens. Matter Mater. Phys.* **2015**, *92*, 184108.
